# Advancing the Understanding of Malnutrition in the Elderly Population: Current Insights and Future Directions

**DOI:** 10.3390/nu16152502

**Published:** 2024-07-31

**Authors:** Anna Tomasiewicz, Jacek Polański, Wojciech Tański

**Affiliations:** 1 Student Research Club of Surgical Specialties, Faculty of Medicine, Wroclaw Medical University, 50-532 Wrocław, Poland; 2 Department of Internal and Occupational Diseases, Hypertension and Clinical Oncology, Wroclaw Medical University, 50-556 Wrocław, Poland; jacek.polanski@umw.edu.pl; 3 Faculty of Medicine, Wrocław University of Science and Technology, 50-376 Wrocław, Poland

**Keywords:** elderly malnutrition, biomarkers, nutritional assessment, sarcopenia, chronic diseases, multidisciplinary approaches

## Abstract

Malnutrition is a growing public health problem leading to increased morbidity and mortality worldwide. Up to 50% of elderly patients are hospitalized due to this condition. In this review, we focused on analyzing the current diagnostic criteria for malnutrition among the elderly population and proposing promising solutions. Currently used diagnostic methods such as BMI or serum albumin levels are not sufficient to indicate malnutrition, which is affected by many factors, including the number of chronic diseases, multiple medications taken, or physical condition. Moreover, current recommendations are inadequate because they fail to account for various factors such as chronic illnesses, multiple medications, and bodily changes that are crucial in diagnostic evaluations. There is a noticeable gap between these recommendations and actual clinical practice. Nevertheless, developing more precise, non-invasive biomarkers and personalized nutrition strategies has to be explored. One of these strategies we discuss in our review is multidisciplinary approaches that combine nutrition, physical activity, and psychosocial support. Addressing malnutrition among the elderly should rely on standardized protocols and personalized interventions to enhance their nutritional health and overall well-being.

## 1. Introduction

Over the past few decades, malnutrition in older adults has been identified as a challenging health concern associated with not only increased mortality and morbidity but also with physical decline, which affects everyday activities and quality of life in general. It has been gaining recognition as a significant public health problem affecting older adults, indicated by either unintentional weight loss or having a low body mass index, especially in cases of chronic diseases, polypharmacy, and functional impairments [1,2]. Numerous studies have indicated that approximately 20–50% of elderly individuals administered in hospitals suffer from this condition, which is strongly associated with an increase in sickness and death rates, elongated hospitalization periods, increased infection rates, and poorer health outcomes in general [3,4,5]. One of the reasons for the recent increase in cases of malnutrition among older patients is the demographic change in developed countries [6]. Unfortunately, the currently used standards for diagnosing malnutrition among elderly patients remain inadequate and often fail to recognize the early stages of this condition [1,2]. While albumin levels, BMI, and weight loss are currently used for diagnosis, they can be influenced by factors such as inflammation or hydration status, leading to incorrect diagnoses [7,8,9]. This underscores the need for more reliable and specific diagnostic criteria. New criteria for diagnosing malnutrition in the elderly aim to boost precision beyond traditional methods like BMI and serum albumin levels. This can be accomplished by including a comprehensive assessment that takes into account non-invasive biomarkers, functional markers, and personalized nutrition approaches. The aim of this review is to critically evaluate current diagnostic criteria for malnutrition in the elderly and propose future directions for improving its early detection and management.

## 2. Pathophysiology of Malnutrition in the Elderly

Malnutrition in the elderly is caused by a combination of physiological changes, chronic diseases, medication use, psychological factors, and socioeconomic issues. As individuals age, they typically encounter a natural decrease in physiological functions, including diminished appetite, shifts in taste and smell sensations, and reduced digestive efficiency, all of which result in inadequate nutrient intake. Chronic diseases such as cancer, cardiovascular diseases, gastrointestinal disorders, and diabetes further enhance malnutrition by increasing metabolic demands or impairing nutrient absorption. Additionally, polypharmacy, which is the simultaneous use of multiple medicines, can lead to side effects such as loss of appetite or gastrointestinal disturbances, which induce malnutrition [10,11]. Methods to reduce these consequences consist of regular medication assessments, stopping unnecessary medications, and including nutritional evaluations in usual medical practice. Psychological factors, including depression and cognitive decline, are among the other factors that significantly affect dietary habits. Depression often leads to a lack of interest in food, while cognitive impairments cause difficulties in meal preparation or hinder the ability to remember to eat [12]. Social isolation, often seen in older adults, can further reduce food intake due to a lack of social eating opportunities and diminished motivation to prepare meals [13]. Moreover, socioeconomic factors such as limited income, social isolation, and lack of access to nutritious foods interact with physiological changes in the elderly, such as reduced appetite and altered taste, to exacerbate malnutrition. For instance, a low-income elderly person may struggle to afford high-quality food, leading to inadequate nutrient intake that worsens physiological decline. Additionally, inadequate knowledge about proper nutrition and food preparation can contribute to poor dietary choices, further increasing the risk of malnutrition [14]. Cultural and subcultural differences significantly impact the diagnosis and perception of malnutrition. For instance, BMI thresholds for underweight and malnutrition may differ across cultures due to genetic and dietary variations [15]. Additionally, healthcare providers’ diagnostic practices are influenced by their training and the administrative rules of their facilities. It is crucial to develop culturally sensitive diagnostic criteria and training programs that account for these variations to ensure accurate assessment and effective intervention [16].

The combination of these factors leads to a high prevalence of malnutrition in the elderly, which has a significant impact on their health and overall quality of life. Malnutrition can lead to weakened immune function, increased susceptibility to infections, slower wound healing, and higher rates of hospitalization and mortality [17]. Addressing these multifactorial causes through comprehensive, individualized care plans is essential for improving the nutritional status and overall well-being of the elderly population [18].

## 3. Biomarkers of Malnutrition in the Elderly

Biomarkers are essential tools for diagnosing and monitoring malnutrition in the elderly. They can be categorized into anthropometric measures, biochemical markers, inflammatory markers, and functional markers, each providing unique insights into an individual’s nutritional status. In the section below, we will discuss the inadequacies of the currently used biomarkers.

### 3.1. Anthropometric Measures

Anthropometric measures, including body mass index (BMI), mid-upper arm circumference (MUAC), and skinfold thickness, are commonly used to assess nutritional status by estimating body composition and fat stores [19]. BMI, which is calculated as weight in kilograms divided by height in meters squared, is a widely utilized indicator due to its simplicity and ease of measurement. However, these measures have significant limitations, particularly in the elderly population [20]. Age-related changes in body composition, such as the loss of muscle mass (sarcopenia) and the redistribution of fat, can lead to inaccurate assessments when using BMI. For instance, an elderly individual with a normal BMI might still have significant muscle wasting and fat redistribution, masking underlying malnutrition. Similarly, MUAC and skinfold thickness can be influenced by changes in skin elasticity and muscle atrophy, common in older adults, leading to a potential misclassification of nutritional status [21,22]. These inadequacies highlight the need for more precise and reliable diagnostic criteria that account for the physiological changes associated with aging, thereby improving the early detection and management of malnutrition in the elderly.

### 3.2. Biochemical Markers

Biochemical markers commonly used to assess malnutrition in the elderly include serum albumin, prealbumin, transferrin, and retinol-binding protein [23]. These markers provide valuable information about protein levels and nutritional status. Serum albumin, a protein made by the liver, is often used as an indicator of long-term nutritional status, while prealbumin and transferrin are used to assess short-term changes due to their shorter half-lives. Retinol-binding protein, which transports vitamin A in the blood, also serves as a marker for nutritional assessment. However, these biochemical markers have significant limitations in the elderly population. Their levels can be influenced by factors other than nutritional intake, such as inflammation, infection, hydration status, liver function, and the presence of chronic diseases [23,24]. For instance, low serum albumin levels may indicate acute or chronic inflammation rather than malnutrition, leading to a potential misdiagnosis. Additionally, the elderly often experience multiple comorbidities and polypharmacy, which can further affect the accuracy of these markers [11]. As a result, relying solely on these biochemical markers can lead to false positives or negatives, underscoring the need for more specific and sensitive biomarkers that can accurately reflect nutritional status without being confounded by other age-related physiological changes. Recent studies highlight that amino acid profiles, particularly levels of tyrosine (Tyr), tryptophan (Trp), and phenylalanine (Phe), can serve as potential biomarkers for malnutrition [25,26], offering more accurate assessments compared to traditional markers like BMI and serum albumin. These amino acids were found to be significantly lower in malnourished individuals compared to those with normal nutritional status.

### 3.3. Inflammatory Markers

Inflammatory markers such as C-reactive protein (CRP), interleukins (e.g., IL-6), and tumor necrosis factor-alpha (TNF-α) are commonly used to assess malnutrition in the elderly [27]. These markers are indicative of the body’s inflammatory response, which can significantly impact nutritional status. Elevated levels of CRP, for instance, are associated with acute inflammation and can signal underlying infections or chronic diseases that often accompany malnutrition [27]. Interleukins and TNF-α are cytokines that play key roles in the inflammatory process and are also elevated in states of chronic inflammation. However, the use of these inflammatory markers in assessing malnutrition in the elderly has notable limitations. Inflammation itself can lead to changes in protein metabolism and nutrient utilization, making it challenging to distinguish whether altered levels are due to malnutrition or an inflammatory response [23,28]. Additionally, chronic low-grade inflammation is common in the elderly due to aging and various comorbidities, which can lead to persistently elevated inflammatory markers independent of nutritional status [29]. This overlap complicates the interpretation of these markers, potentially leading to misdiagnosis or underestimation of malnutrition. Given the limitations of inflammatory markers like CRP in distinguishing between inflammation and malnutrition, alternative approaches such as the combined use of inflammatory and non-inflammatory biomarkers or the development of more specific markers like certain cytokine profiles are suggested. Additionally, advanced imaging techniques and omics technologies can provide more detailed insights [30]. Therefore, while inflammatory markers are valuable in providing a broader picture of an elderly patient’s health status, they must be used in combination with other diagnostic tools to accurately assess malnutrition without being confounded by the multifaceted nature of aging and disease.

### 3.4. Functional Markers

Functional markers, such as handgrip strength, gait speed, and activities of daily living (ADLs), are commonly used to evaluate malnutrition in older adults [31,32]. Handgrip strength, measured by a dynamometer, provides a quick and practical assessment of muscle function and overall nutritional status. Gait speed, on the other hand, assessed by timing a short walk, is a reflection of physical performance and can predict adverse health outcomes. ADLs, which include basic self-care activities like bathing, dressing, and eating, offer insights into an individual’s functional independence and nutritional well-being. However, these functional markers also have limitations in accurately diagnosing malnutrition in the elderly. Age-related declines in muscle mass and function, known as sarcopenia, can affect these markers, making it difficult to distinguish between malnutrition and normal aging [33]. To differentiate the effects of sarcopenia from malnutrition using functional markers like handgrip strength and gait speed, it is crucial to incorporate additional assessments such as muscle mass measurement through DXA scans or bioelectrical impedance analysis [34,35]. These assessments, combined, can help distinguish between muscle wasting due to sarcopenia and overall nutritional deficits. Additionally, chronic illnesses, cognitive impairments, and comorbidities prevalent in older adults can influence these functional measures, leading to a potential misinterpretation of nutritional status [36]. Therefore, while functional markers are valuable for assessing the impact of malnutrition on physical capabilities, they must be used alongside other diagnostic tools to provide a comprehensive evaluation of an elderly person’s nutritional health.

Recent systematic reviews and meta-analyses have evaluated various biomarkers associated with malnutrition risk in older adults. Key findings suggest that BMI, hemoglobin, and total cholesterol are useful biomarkers, with serum albumin and prealbumin being particularly sensitive indicators, though their levels can be significantly affected in acute illness settings [23]. Advanced techniques such as targeted metabolomics are also emerging, offering a more nuanced understanding of the biochemical changes associated with malnutrition [26]. In summary, a comprehensive approach using a combination of these biomarkers is recommended for accurately diagnosing and managing malnutrition in the elderly. Integrating advanced technologies and personalized nutrition strategies may further enhance the effectiveness of these assessments. Dietary recommendations and interventions can be customized based on an individual’s specific genetic composition, metabolic profile, and health needs.

## 4. Current Knowledge and Clinical Guidelines

The currently described guidelines focus on nutrition plans that are directly adjusted to individuals, specified based on their needs, and include other factors such as comorbidities, medication interactions, and overall health status [37]. Nutritional aspects are significantly important to reduce the chances of malnutrition. The earlier we raise awareness about the consequences of malnutrition, the less likely we are to suffer from this dietary dysfunction. Regular and systematic monitoring is crucial to providing the correct nutritional intervention and allowing necessary adjustments. These interventions should be implemented in clinical practice and routinely used [37].

Major organizations, including the European Society for Clinical Nutrition and Metabolism (ESPEN), are used around the world to provide clinical guidelines to assess and manage malnutrition among the elderly population. These guidelines include the outlines for routine screening, accurate assessment, and personalized intervention plans. The 2023 ESPEN guidelines that are followed in European countries recommend routine screening for malnutrition using tools such as the Mini Nutritional Assessment (MNA) [38] and the Malnutrition Universal Screening Tool (MUST) [39]. These tools play a beneficial role in identifying people at increased risk of malnutrition and allowing early intervention to reduce the potential pain caused by this health problem. These guidelines include strategies for proper measurements, including anthropometric data and biochemical markers, to increase the rate of correct diagnosis of malnutrition [37].

Biochemical markers recommended by the current standards include serum albumin, prealbumin, transferrin, and retinol-binding protein [24]. However, their specificity can be influenced by the impact of inflammation and fluid accumulation. Therefore, these markers should be combined with functional assessments, such as handgrip strength and gait speed, to offer a more rational view of the patient’s nutritional status [37].

One of the frameworks consisting of phenotypic and etiologic criteria to diagnose malnutrition is the Global Leadership Initiative on Malnutrition (GLIM) criteria described in the 2023 ESPEN guidelines [40], which suggest a two-step approach. At first, it is crucial to identify at-risk individuals through precise screening. Subsequently, the next step is to diagnose malnutrition status based on phenotypic criteria (e.g., weight loss, low BMI, reduced muscle mass) and etiologic criteria (e.g., reduced food intake or assimilation, inflammation). This approach helps in characterizing a patient as malnourished and allows for appropriate nutritional interventions [40].

Several recent systematic reviews and meta-analyses have thoroughly provided a detailed examination of the prevalence, causes, and management of malnutrition among older adults. Dent et al. (2023) highlighted that routine screening and nutritional assessment, followed by individualized nutritional support, are required to assess the malnutritional status of GP practitioners [1]. Nutrition conditions may be assessed through dietary counseling, oral nutritional supplements, fortified foods, and enteral or parenteral nutrition when necessary. Despite established guidelines, there is a significant gap between evidence-based recommendations and clinical practice, often resulting in inadequate care. To bridge this gap, practical strategies may be proposed, including the integration of nutritional guidelines into quality assurance frameworks and emphasizing the need for person-centered, feasible approaches to nutritional care for older adults [1].

Another factor affecting malnutrition status is the overall physical condition. This factor was investigated in more detail in a comprehensive meta-analysis aggregating data from 45 observational studies [41]. A total of 16,911 participants were included in this analysis. This study revealed that malnourished and at-risk older adults exhibited significantly poorer physical performance compared to their well-nourished counterparts. The weakness of malnourished participants was indicated by key measurements of physical activity, including hand grip strength, gait speed, and the timed-up-and-go test. This study suggests the need for more standardized and controlled protocols that may be useful to evaluate constant measurements that can be applied to every individual to correctly determine malnutrition status [41].

The presence of other secondary diseases also affects the malnutrition status. This is the case of elderly patients suffering from cancer, as described in the Bullock et al., study [42]. Approximately 15 different markers of nutritional status were evaluated among 21,032 participants aged 70 or older. The study revealed that decreased food intake was significantly correlated with higher mortality rates. In contrast, the presence of the Prognostic Nutritional Index (PNI), including markers like albumin and total lymphocyte count, was associated with overall survival. To sum up, these findings prove the need for improved nutritional assessments and interventions to enhance the clinical management of older cancer patients [42].

Another study aimed to evaluate the association between blood biomarkers and malnutrition risk in older adults [23]. Their findings were specific to albumin, hemoglobin, total cholesterol, and prealbumin. All these biomarkers show significantly lower amounts among individuals at high risk of malnutrition. It is worth noting that although albumin and prealbumin are commonly used biomarkers of malnutrition, their levels can be additionally affected by acute inflammatory stress, which should be considered while interpreting the clinical results by GP practitioners. These findings confirm the role of developing and implementing updated reference ranges and cut-off values to improve the accuracy of malnutrition diagnosis in older adults [23].

Chuansangeam et al., reviewed the cumulative work of 71 studies with a total of 23,788 individuals aged 60 and above living in Thailand [43]. They aimed to investigate differences between the malnutrition status of hospitalized patients and that of community-dwelling elders. In accordance with Besora-Moreno’s study [14], they also listed significant sociodemographic factors affecting malnutrition status, including female sex, advanced age, low education, living alone, and rural residency. Their main finding was that the pooled prevalence of malnutrition was higher in hospitalized patients compared to community-dwelling elders. Other than sociodemographic factors, there were other comorbidities and geriatric conditions, such as dementia and depression. These results are additional examples emphasizing the need for targeted nutritional interventions and community-directed food systems to mitigate malnutrition among older adults in Thailand [43].

Consistent with the previously discussed study by Chuansangeam et al., female sex was also a sociodemographic factor increasing the risk of malnutrition in India [44]. This study combined the work of 45 studies between 2010 and 2019 and consisted of 10,518 participants. The findings indicated that the pooled prevalence of malnutrition among the elderly was 18.29%, while nearly half were at risk of malnutrition. Other than the female sex factors listed in this study, those living in urban areas had significant regional variations observed, particularly in Northern India. These results underscore the urgent need for targeted nutritional interventions and policies to address malnutrition among the elderly, particularly in high-risk settings such as old age homes and clinical environments [44].

Furthermore, in line with Chuansangeam et al. [43], low education and living alone increase the likelihood of suffering from malnutrition, especially among older adults. This was tested by Besora-Moreno et al., who reviewed 40 observational studies from different countries, including 34 cross-sectional and four cohort studies [14]. A total of 34,703 individuals aged 60 and older were included in this review study. The other sociodemographic factors associated with a high risk of malnutrition are being single, widowed, or divorced, and having a low income. These findings clearly implicate the necessity of targeted interventions to address socioeconomic disparities and improve nutritional outcomes in older adults. These strategies may enhance economic levels, provide support for those living alone, and promote lifelong learning among the elderly population [14].

One of the main strategies to indirectly progress the management of malnutrition among community-dwelling older adults is telehealth intervention. The effectiveness of this method was reviewed by Marx et al., who combined the results of nine studies; all of them demonstrated improved protein intake and overall wellbeing among participants after telehealth consultations [45]. Although the scale of help delivered from telehealth interventions with patients ranged from low to very low, it is still a promising direction to follow to deliver nutritional support to older adults. In consequence, this strategy may provide clinical improvements such as better nutritional status, physical function, and potentially reduced hospital readmission rates and mortality. The main limitation of these studies is the low number of participants involved. However, telehealth interventions are potentially a good sign to provide nutritional support for elderly populations, especially because of the increased awareness of the usage of novelties of modern civilization among older adults, such as smartphones, social media on laptops, etc.

Many different factors should be considered while deciding whether the patient is hospitalized due to malnutrition conditions or not. Dent et al., highlighted some of these factors, including inadequate meal services, inflexible mealtimes, and insufficient energy content in hospital meals [46]. Furthermore, this study also pointed out the significant role of medical staff, who are on the first line to obtain an impression about the nutritional status of hospitalized patients. A total of 32 nutritional screening tools (NSTs) used in hospitals were reviewed for their validity and reliability. About 23 of them were specifically designed for the elderly population. Although many different screening tools are used, there is limited cooperative data about their effectiveness for the elderly population. The conclusion of this study was the importance of the development of biomarkers that play a role in the early identification of malnutrition. This may be useful to improve clinical outcomes through reduced mortality, functional decline, or prolonged hospital stays. This comprehensive insight into NSTs aimed to bridge the gap between the high prevalence of malnutrition and the often inadequate recognition and treatment within hospital environments.

## 5. Current Limitations and Future Perspectives

Although the growing interest in science on malnutrition among the elderly in recent years has been observed, some limitations still exist and contribute to the erroneous perception of malnutrition. Inconsistency in the use of biomarkers is a significant problem. Many healthcare facilities use different protocols. Therefore, the lack of validated standard protocols affects the quality of malnutrition treatment and the correct diagnosis.

It is very likely to obtain false positive or negative results if biomarkers are chosen without considering other factors than nutritional ones, such as inflammation or the patient’s hydration. The presence of comorbidities and semi-gravity on the nutritional status should also be taken into account. Lack of knowledge in this area due to incorrectly conducted medical consultations complicates the assessment and treatment of malnutrition.

The co-occurrence of malnutrition and sarcopenia, known as the malnutrition-disability cycle, presents a particular challenge. This cycle describes the clinical presentation of malnutrition along with accelerated age-associated loss of lean body mass, strength, and functionality. Research indicates that this combination worsens the prognosis for elderly patients, yet there remains a lack of clear guidelines on how to effectively diagnose and manage these coexisting conditions [33,47].

Developing more precise and non-invasive biomarkers, such as blood-based markers, urine tests, or salivary diagnostics, allows for the detection of nutritional deficiencies and metabolic changes without the need for invasive procedures. These promising biomarkers, which could detect malnutrition at earlier stages than those currently used, should be considered from further perspectives. Age-related changes in metabolic profiles can be quantitatively assessed, and the analytical techniques used for metabolite detection are highly sensitive and specific [30]. However, further studies are needed to evaluate the sensitivity and specificity of the proposed multiomic biomarkers for diagnosing malnutrition in the elderly [48]. In order to properly assess nutritional status, novel biomarkers should also be included in the package of nutritional approaches directed to individuals, as well as genetic, metabolic, and environmental. These approaches should be adjusted to personal needs to correctly improve the quality of life among the elderly population.

Furthermore, research should evaluate the efficacy of personalized nutrition interventions in improving health outcomes in the elderly. For instance, a customized nutrition plan may comprise personalized meal plans that account for particular nutrient deficiencies detected through biomarker testing, thereby enhancing nutritional intake and dealing with individual health issues more effectively. This includes clinical trials comparing personalized approaches to standard care, such as stroke, cancer, or orthopedic injuries like a broken leg. It is crucial to take into consideration the various nutritional backgrounds patients may come from when thinking about institutional meals, particularly in hospitals. The settings can change significantly based on the medical issues of the elderly patient, like stroke, cancer, or orthopedic injuries such as a broken leg. Last but not least, scientists should investigate the benefits of integrated, multidisciplinary approaches to managing malnutrition, combining nutritional support with physical activity and psychosocial interventions [41,49]. Public health practitioners should focus on the early detection and management of malnutrition, likely through the use of proper biomarkers. It can only be mediated through the cooperative work of scientists, clinicians, and policymakers.

Last but not least, novel technologies such as m-health services, enhanced by artificial intelligence (AI), offer significant advancements in the automatic, objective, and continuous monitoring of malnutrition risk, making early detection and management more effective and trustworthy through explainable AI (XAI) methodologies [50]. However, despite significant advancements, over 90% of DSS solutions have not been implemented in practice, highlighting a critical gap between research and practical application and identifying key challenges for future exploration and maturation of these systems [51].

To sum up, although many scientific interventions have already been demonstrated to improve knowledge about management, some significant gaps remain to be filled. To address these challenges, researchers must continuously work together with the development of modern technologies and a collaborative approach across various healthcare and policy domains.

## 6. Conclusions

Malnutrition among older adults has a negative impact on their quality of life and affects healthcare costs. Although a vast majority of studies have already been reported regarding the prevalence, causes, and effects of malnutrition in the elderly population, biochemical markers and their role in diagnosing malnutrition levels still need to be investigated in detail. Currently used biomarkers are categorized into anthropometric measures, biochemical markers, inflammatory markers, and functional assessments. Each of them has several valuable advantages, but several limitations cannot be omitted. To date, there is no perfect single biomarker as a tool for diagnosing and monitoring malnutrition among the studied population.

The complexity of malnutrition is influenced by physiological changes, chronic diseases, medications, and socioeconomic factors; hence, multiple approaches are required to assess malnutrition conditions correctly. As the outlook for further development in this area grows, more precise, non-invasive, and comprehensive biomarkers demonstrating nutritional status and suggesting potential outcomes should be investigated. Nutritional approaches directed at individuals, together with modern techniques, are promising strategies for the effective management of malnutrition among older adults.

A cooperative effort from researchers, GP practitioners, and policymakers is crucial to prioritizing the design and generation of standardized protocols and multidisciplinary interventions. By advancing our understanding, we can benefit from our work to effectively support the nutritional health and overall well-being of the older population.

## Data Availability

No new data were created or analyzed in this study. Data sharing is not applicable to this article.
